# Metabolic Abnormalities Are Common among South American Hispanics Subjects with Normal Weight or Excess Body Weight: The CRONICAS Cohort Study

**DOI:** 10.1371/journal.pone.0138968

**Published:** 2015-11-23

**Authors:** Catherine P. Benziger, Antonio Bernabé-Ortiz, Robert H. Gilman, William Checkley, Liam Smeeth, Germán Málaga, J. Jaime Miranda, CRONICAS Cohort Study group

**Affiliations:** 1 CRONICAS Center of Excellence in Chronic Diseases, Universidad Peruana Cayetano Heredia, Lima, Peru; 2 Division of Cardiology, University of Washington, Seattle, WA, United States of America; 3 Program in Global Disease Epidemiology and Control, Department of International Health, Bloomberg School of Public Health, Johns Hopkins University, Baltimore, MD, United States of America; 4 Asociación Benéfica PRISMA, Lima, Peru; 5 Division of Pulmonary and Critical Care, School of Medicine Johns Hopkins University, Baltimore, MD, United States of America; 6 Faculty of Epidemiology and Population Health, London School of Hygiene and Tropical Medicine, London, United Kingdom; 7 Facultad de Medicina “Alberto Hurtado”, Universidad Peruana Cayetano Heredia, Lima, Peru; University of the Balearic Islands, SPAIN

## Abstract

**Objective:**

We aimed to characterize metabolic status by body mass index (BMI) status.

**Methods:**

The CRONICAS longitudinal study was performed in an age-and-sex stratified random sample of participants aged 35 years or older in four Peruvian settings: Lima (Peru’s capital, costal urban, highly urbanized), urban and rural Puno (both high-altitude), and Tumbes (costal semirural). Data from the baseline study, conducted in 2010, was used. Individuals were classified by BMI as normal weight (18.5–24.9 kg/m^2^), overweight (25.0–29.9 kg/m^2^), and obese (≥30 kg/m^2^), and as metabolically healthy (0–1 metabolic abnormality) or metabolically unhealthy (≥2 abnormalities). Abnormalities included individual components of the metabolic syndrome, high-sensitivity C-reactive protein, and insulin resistance.

**Results:**

A total of 3088 (age 55.6±12.6 years, 51.3% females) had all measurements. Of these, 890 (28.8%), 1361 (44.1%) and 837 (27.1%) were normal weight, overweight and obese, respectively. Overall, 19.0% of normal weight in contrast to 54.9% of overweight and 77.7% of obese individuals had ≥3 risk factors (p<0.001). Among normal weight individuals, 43.1% were metabolically unhealthy, and age ≥65 years, female, and highest socioeconomic groups were more likely to have this pattern. In contrast, only 16.4% of overweight and 3.9% of obese individuals were metabolically healthy and, compared to Lima, the rural and urban sites in Puno were more likely to have a metabolically healthier profile.

**Conclusions:**

Most Peruvians with overweight and obesity have additional risk factors for cardiovascular disease, as well as a majority of those with a healthy weight. Prevention programs aimed at individuals with a normal BMI, and those who are overweight and obese, are urgently needed, such as screening for elevated fasting cholesterol and glucose.

## Introduction

Obesity, commonly measured using body mass index (BMI), has been associated with a number of metabolic and cardiovascular disease (CVD) risk factors including an excess mortality risk [1]. Levels of overweight and obesity in Latin America have increased over time [2] and have approached levels found in higher-income countries, with a disproportionate increase in waist circumference compared to BMI over the past 20 years [3, 4]. Overweight and obesity and are projected to continue rising [5].

The World Health Organization (WHO) recommended cut-off points for overweight and obesity, at BMI values of 25 kg/m^2^ and 30 kg/m^2^, respectively, are based on a large number of studies in predominantly Caucasian populations. Prior studies in high-income settings have noted a high prevalence of individuals who are overweight and obese but display normal metabolic features despite their increased adiposity [6–12]. This metabolically healthy obese profile is controversial: whilst some evidence suggests these individuals are still at increased risk of developing diabetes [13, 14], CVD [15, 16] and have increased mortality [8], other studies have found an “obesity paradox” whereby there seems to be a protective effect of obesity from mortality and other chronic conditions [17]. Fasting insulin levels in obese individuals can help further differentiate healthy versus unhealthy as increased levels are associated with development of risk factors for CVD and increased mortality [11, 18, 19]. Conversely, there are also individuals who are normal weight but display metabolically unhealthy features with increased risk of diabetes and CVD [11, 20, 21]. No prior studies have estimated the prevalence of overweight and obese individuals and their metabolic risk factors and if these individuals are different with regards to socio-demographic and behavioral factors in a Latin American population.

As a rapid nutritional transition occurs in Peru [22] and the prevalence of obesity continues to increase, it is unknown to what extent these profiles exist in this population and whom to target with public health interventions at the community health worker level. Peru also has geographical diversity; a pattern shared with other Latin American countries, and differences in these locations is unknown. Based on BMI, prior studies in Peru have reported a prevalence of 40% and 15–23% for overweight and obesity, respectively [23, 24], with increased odds of obesity among those who are older and female [24, 25]. The prevalence of metabolic syndrome is between 17–25%, depending on which definition is used (American Heart Association or International Diabetes Federation) [26, 27]. However, none have evaluated the prevalence of metabolically healthy obese or metabolically unhealthy normal weight individuals in different settings in Peru. Therefore, we hypothesized that the metabolically healthy obese group compared to the healthy normal weight group was different with regards to socio-demographic and behavioral factors in a Latin American population. In addition, we hypothesized that the metabolically unhealthy normal weight group compared to the metabolically unhealthy overweight and obese group had different socio-demographic and behavior factors. Given our hypotheses, we aimed to use an established definition of metabolic status and to do the following: 1) estimate the prevalence of each of the BMI categories (normal weight with and without metabolic abnormalities, and overweight and obese with and without metabolic abnormalities); 2) determine the prevalence of metabolically healthy obese according to socio-demographic and behavioral factors; and 3) determine the prevalence of unhealthy metabolic status if normal weight according to socio-demographic and behavioral factors.

## Methods

### Study Design, Setting and Participants

The objectives and design of the CRONICAS cohort study have been reported elsewhere [28]. Briefly, a longitudinal study was performed in an age-and-sex stratified random sample of participants aged 35 years or older in four Peruvian settings: Lima (Peru’s capital, costal urban, highly urbanized), urban and rural Puno (both high-altitude), and Tumbes (costal semirural). Data from the baseline study, conducted in 2010, was used for this study and analysis was completed in 2014.

### Data Collection

A team of community health workers was trained to enroll participants and to conduct the questionnaires assessing socio-demographic and behavioral variables (Table 1). Variables included sex, age, study site, education, and socioeconomic status, the latter constructed from the aggregation of assets and household facilities into a wealth index split in tertiles [29]. Behavioral risk factors included were: current daily smoking, hazardous alcohol drinking [30], and leisure time and transport-related physical activity (Table 1).

**Table 1:** Definition of sociodemographic, behavioral and metabolic variables.

**A. Sociodemographic and behavioral factors considered:**
**Age groups**: 35–44, 45–54, 55–64, ≥65 years
**Education:** none or primary (<6 years), secondary (7–11 years) and superior (12 or more years)
**Wealth Index:** based on assets and household facilities divided into tertiles.
**Site**: Lima (costal urban), Puno urban (high-altitude urban), Puno rural (high-altitude rural), and Tumbes (costal rural)
**Daily smoking:** ≥1 cigarette/day, self-report.
**Alcohol drinking:** The Alcohol Use Disorders Identification Test [AUDIT] score ≥8 points for hazardous drinking [30].
**Leisure-time physical activity:** Based on leisure time domain of IPAQ as recommended for Latin American populations [31]. Categories of levels of physical activity were coded based on both total days of physical activity.
**Transport-related physical activity:** Based on transport time domain of IPAQ as recommended for Latin American populations [31]. Categories of levels of physical activity were coded based on both total days of physical activity.
B. Metabolic abnormalities considered*:
**Elevated blood pressure:** systolic blood pressure ≥130mmHg, diastolic blood pressure ≥85mmHg, antihypertensive medication, or history of hypertension
**Elevated triglyceride level:** fasting triglyceride level ≥150 mg/dL
**Decreased HDL-C level:** HDL-C level <40 mg/dL in men <50 mg/dL in women
**Elevated glucose level:** fasting glucose level ≥100 mg/dL or glucose-lowering medication
**Waist circumference:** ≥90 cm in men or ≥80 cm in women
**Insulin resistance:** HOMA-IR >5.00 (i.e. >90th percentile)
**Systemic inflammation:** hs-CRP level > 7.11mg/L (i.e. >90th percentile)
**C. Criteria for body size and metabolic profiles:**
Normal weight, metabolically healthy: BMI 18.5–24.9 and <2 risk factors
Normal weight, metabolically unhealthy: BMI 18.5–24.9 and ≥2 risk factors
Overweight, metabolically healthy: BMI 25.0–29.9 and <2 risk factors
Overweight, metabolically unhealthy: BMI 25.0–29.9 and ≥2 risk factors
Obese, metabolically healthy: BMI ≥30.0 and <2 risk factors
Obese, metabolically unhealthy: BMI ≥30.0 and ≥2 risk factors

* Based on the International Diabetes Federation’s definition of metabolic syndrome [32] and Wildman *et al* [7].

Abbreviations: METs, metabolic equivalent tasks; HDL-C, high-density lipoprotein cholesterol; HOMA-IR, homeostasis model assessment of insulin resistance; hsCRP, high-sensitivity C-reactive protein; BMI, body mass index (calculated as weight in kilograms divided by height in meters squared).

Participants were invited to a clinic visit where height, weight, waist circumference, and systolic (SBP) and diastolic (DBP) blood pressure, as well as fasting blood samples were obtained using standardized methods and calibrated tools [28]. Total cholesterol, triglycerides, high-density lipoprotein cholesterol (HDL-c), insulin, and high-sensitivity C-reactive protein (hs-CRP) were measured in serum, whereas fasting glucose was assessed in plasma.

### Body Size Definitions

Subjects were classified based on BMI as normal weight (18.5–24.9 kg/m^2^), overweight (25.0–29.9 kg/m^2^), and obese (≥30 kg/m^2^).

### Metabolic Status Definition

Metabolic syndrome and its components were defined according to International Diabetes Federation criteria [32] (Table 1): waist circumference cutoffs (≥90 cm in men or ≥80 cm in women) for South Asian individuals was used as recommended; fasting triglyceride level ≥150 mg/dL; HDL-c level <40 mg/dL in men <50 mg/dL in women; systolic blood pressure ≥130mmHg, diastolic blood pressure ≥85mmHg, antihypertensive medication, or history of hypertension [33]; fasting glucose level ≥100 mg/dL or glucose-lowering medication. Elevated hs-CRP (>7.11mg/dL, >90^th^ percentile) and homeostasis model assessment of insulin resistance value (HOMA-IR) (>5.00, >90^th^ percentile) were used using cut-offs proposed previously by Wildman et al. [7]. We classified individuals into three risk groups: 0 or 1 abnormal risk factor, 2 abnormal risk factors, and ≥3 abnormal risk factors by BMI. For the regression analysis, individuals were classified as metabolically healthy (0 or 1 abnormal risk factor) or unhealthy (≥2 abnormal risk factors), as previously defined [7, 8].

### Statistical Analysis

Initially, a description of the socio-demographic, behavioral and clinical variables overall and according to BMI and metabolic status was performed. Participants that did not have all of the metabolic factors measured were excluded from the analysis. Geometric means were calculated for non-normally distributed continuous variables. Differences in these variables among the three categories (normal weight, overweight, and obese) were analyzed within each metabolic subgroup using Chi-squared test and one-way analysis of variance.

Among normal weight individuals, prevalence ratios of being metabolically unhealthy were calculated using socio-demographic and behavioral variables in unadjusted models. Subsequently, we used multivariable generalized linear models assuming Poisson distribution of the outcome and robust standard errors to obtain prevalence ratios (PR) and 95% confidence intervals (95%CI), adjusting for all the variables simultaneously, without and with waist circumference into the model. Among overweight and obese individuals, prevalence ratios of being metabolically healthy were calculated using socio-demographic and behavioral variables in unadjusted models and subsequently in multivariable generalized linear models as stated previously. STATA 10 (STATA CORP, College Station, Texas, USA) was used for all analyses.

### Ethics

The study was approved by the Institutional Review Boards at Universidad Peruana Cayetano Heredia and A.B. PRISMA, in Lima, Peru, and at the Bloomberg School of Public Health, Johns Hopkins University, in Baltimore, USA. All participants provided verbal informed consent after our research team read the entire informed consent document to them and any questions were answered. Informed consents were verbal because of high illiteracy rates. Both ethics committees approved the verbal consent procedure.

## Results

A total of 3088 (85.4%) of 3618 subjects had all the factors measured. The sample had 51.3% females and on average the sample age was 55.6 years (SD ±12.6 years). Based on BMI, 890 subjects (28.8%) were normal weight, 1361 (44.1%) were overweight, and 837 (27.1%) were obese.

### Metabolic Risk Profiles

Baseline socio-demographic, behavioral, and clinical variables overall and by BMI categories and metabolic status are shown in Table 2. Each of the five components of metabolic syndrome was prevalent in at least 25% of the population. A total of 2263 (73.3%) had elevated waist circumference, 2048 (66.4%) had low HDL-c, 1329 (43.1%) had elevated triglycerides, 893 (28.9%) had elevated blood pressure, and 798 (25.9%) had impaired fasting glucose.

**Table 2:** Baseline demographic and metabolic characteristics of the study population by body size.

	Metabolically healthy (N = 763)				Metabolically unhealthy (N = 2324)			
	Normal weight	Overweight	Obese	p-value	Normal weight	Overweight	Obese	p-value
	N (%)	N (%)	N (%)		N (%)	N (%)	N (%)	
**Prevalence**	507 (66.5)	223 (29.2)	33 (4.3)		383 (16.5)	1138 (48.9)	804 (34.6)	
**Sociodemographic characteristics**								
*** Age groups***				0.002				<0.001
35–44 years	122 (24.1)	75 (33.6)	9 (27.3)		60 (15.7)	306 (26.9)	185 (23.0)	
45–54 years	125 (24.7)	64 (28.7)	9 (27.3)		74 (19.3)	290 (25.5)	228 (28.4)	
55–64 years	109 (21.5)	52 (23.3)	8 (24.2)		88 (23.0)	291 (25.6)	235 (29.2)	
≥65 years	150 (29.6)	32 (14.4)	7 (21.2)		161 (42.0)	250 (22.0)	156 (19.4)	
***Female sex***	193 (38.1)	76 (34.1)	22 (66.7)	0.002	199 (52.0)	563 (49.5)	530 (65.9)	<0.001
***Study site***				<0.001				<0.001
Lima	131 (25.8)	81 (36.3)	10 (30.3)		103 (26.9)	383 (33.7)	317 (39.5)	
Urban Puno	70 (13.8)	54 (24.2)	8 (24.2)		51 (13.3)	204 (17.9)	124 (15.4)	
Rural Puno	194 (38.3)	38 (17.1)	3 (9.1)		92 (24.0)	153 (13.4)	50 (6.2)	
Tumbes	112 (22.1)	50 (22.4)	12 (36.4)		137 (35.8)	398 (35.0)	313 (38.9)	
***Education level***				<0.001				<0.001
None or primary	255 (50.3)	74 (33.3)	14 (42.4)		220 (57.4)	465 (40.9)	384 (47.8)	
Secondary	180 (35.5)	88 (39.6)	9 (27.3)		95 (24.8)	378 (33.3)	267 (33.2)	
Superior	72 (14.2)	60 (27.1)	10 (30.3)		68 (17.8)	294 (25.9)	153 (19.0)	
***Socioeconomic status***				<0.001				<0.001
Lowest (poorest)	243 (47.9)	45 (20.2)	8 (24.2)		156 (40.7)	313 (27.5)	189 (23.5)	
Middle	165 (32.5)	85 (38.1)	13 (39.4)		119 (31.1)	382 (33.6)	304 (37.8)	
Highest (richest)	99 (19.5)	93 (41.7)	12 (36.4)		108 (28.2)	443 (38.9)	311 (38.7)	
**Behavioral factors**								
***Smoking***				0.38				0.20
Current daily smoking	20 (3.9)	6 (2.7)	0 (0.0)		17 (4.4)	38 (3.3)	20 (2.5)	
***Alcohol drinking (AUDIT)***				0.07				0.14
Hazardous drinking	85 (16.8)	50 (22.4)	3 (9.1)		50 (13.1)	150 (13.2)	83 (10.3)	
***Physical activity (PA)***								
Low leisure-time PA	479 (94.5)	208 (93.3)	32 (97.0)	0.64	367 (95.8)	1069 (93.9)	771 (95.9)	0.10
Low transport-related PA	154 (30.4)	64 (28.7)	11 (33.3)	0.82	168 (44.0)	392 (34.5)	309 (38.4)	0.003
**Metabolic factors**								
***BMI (kg/m*** ^***2***^ ***)***	22.5 (1.6)	26.8 (1.3)	32.6 (2.4)	<0.001	23.2 (1.5)	27.6 (1.4)	33.5 (3.3)	<0.001
***Abdominal obesity*, *%***	42 (8.3)	124 (55.6)	33 (100.0)	<0.001	198 (51.7)	1063 (93.4)	804 (100.0)	<0.001
***SBP (mmHg)***	111.5 (16.4)	110.9 (11.2)	111.9 (11.4)	0.87	122.0 (21.5)	118.5 (18.9)	120.5 (19.9)	0.004
***DBP (mmHg)***	69.3 (9.4)	70.0 (7.6)	71.3 (6.4)	0.30	74.0 (12.4)	73.8 (10.6)	76.4 (12.0)	<0.001
***Elevated blood pressure*, *%***	58 (11.4)	11 (4.9)	0 (0.0)	0.003	168 (43.9)	353 (31.0)	303 (37.7)	<0.001
***HDL-cholesterol (mg/dL)***	50.2 (11.7)	49.9 (10.5)	54.9 (9.8)	0.05	41.4 (10.5)	38.2 (9.6)	37.8 (9.6)	<0.001
***Low HDL-cholesterol*, *%***	126 (24.9)	30 (13.5)	0 (0.0)	<0.001	284 (74.2)	924 (81.2)	685 (85.2)	<0.001
***Triglycerides (mg/dL)***	96.9 (34.6)	102.5 (34.0)	105.9 (25.4)	0.06	158.6 (92.0)	182.9 (112.0)	188.6 (108.6)	<0.001
***High triglycerides*, *%***	24 (4.7)	9 (4.0)	0 (0.0)	0.42	172 (44.9)	644 (56.6)	481 (59.8)	<0.001
***Fasting glucose (mg/dL)***	86.6 (12.7)	88.7 (6.9)	87.6 (6.8)	0.07	99.6 (37.6)	99.8 (36.9)	105.7 (39.0)	0.001
***Impaired fasting glucose*, *%***	21 (4.1)	5 (2.2)	0 (0.0)	0.23	106 (27.7)	336 (29.5)	330 (41.0)	<0.001
***Insulin (µU/ml)***	4.2 (2.5)	6.3 (3.5)	9.6 (4.3)	<0.001	6.5 (4.7)	9.9 (6.8)	15.9 (12.6)	<0.001
***HOMA-IR***	0.9 (0.6)	1.4 (0.8)	2.1 (0.9)	<0.001	1.6 (1.6)	2.5 (2.3)	4.3 (4.1)	<0.001
***HOMA-IR > 90*** ^***th***^ ***percentile*, *%***	0 (0.0)	0 (0.0)	0 (0.0)	—	13 (3.4)	84 (7.4)	212 (26.4)	<0.001
***High sensitive-CRP (mg/L)***	1.9 (5.5)	1.6 (1.5)	2.5 (1.9)	0.46	4.8 (9.9)	3.6 (8.1)	4.4 (5.2)	0.005
***hs-CRP > 90*** ^***th***^ ***percentile*, *%***	14 (2.8)	1 (0.5)	0 (0.0)	0.08	60 (15.7)	109 (9.6)	125 (15.6)	<0.001

Abbreviations: AUDIT, Alcohol Use Disorders Identification Test; METs, metabolic equivalent tasks; BMI, body mass index (calculated as weight in kilograms divided by height in meters); SBP, systolic blood pressure; DBP, diastolic blood pressure; PA, physical activity; HDL-C, high-density lipoprotein cholesterol; HOMA-IR, homeostasis model assessment of insulin resistance; hs-CRP, high-sensitivity C-reactive protein.

SI conversion factors: To convert to milimoles per liter, multiply by 0.0259 for HDL-C, by 0.0113 for triglycerides, and by 0.0555 for glucose; to convert insulin to picomoles per liter, multiply by 6.945; and hs-CRP to nanomoles per liter, multiply by 9.524.

**Fig 1:**
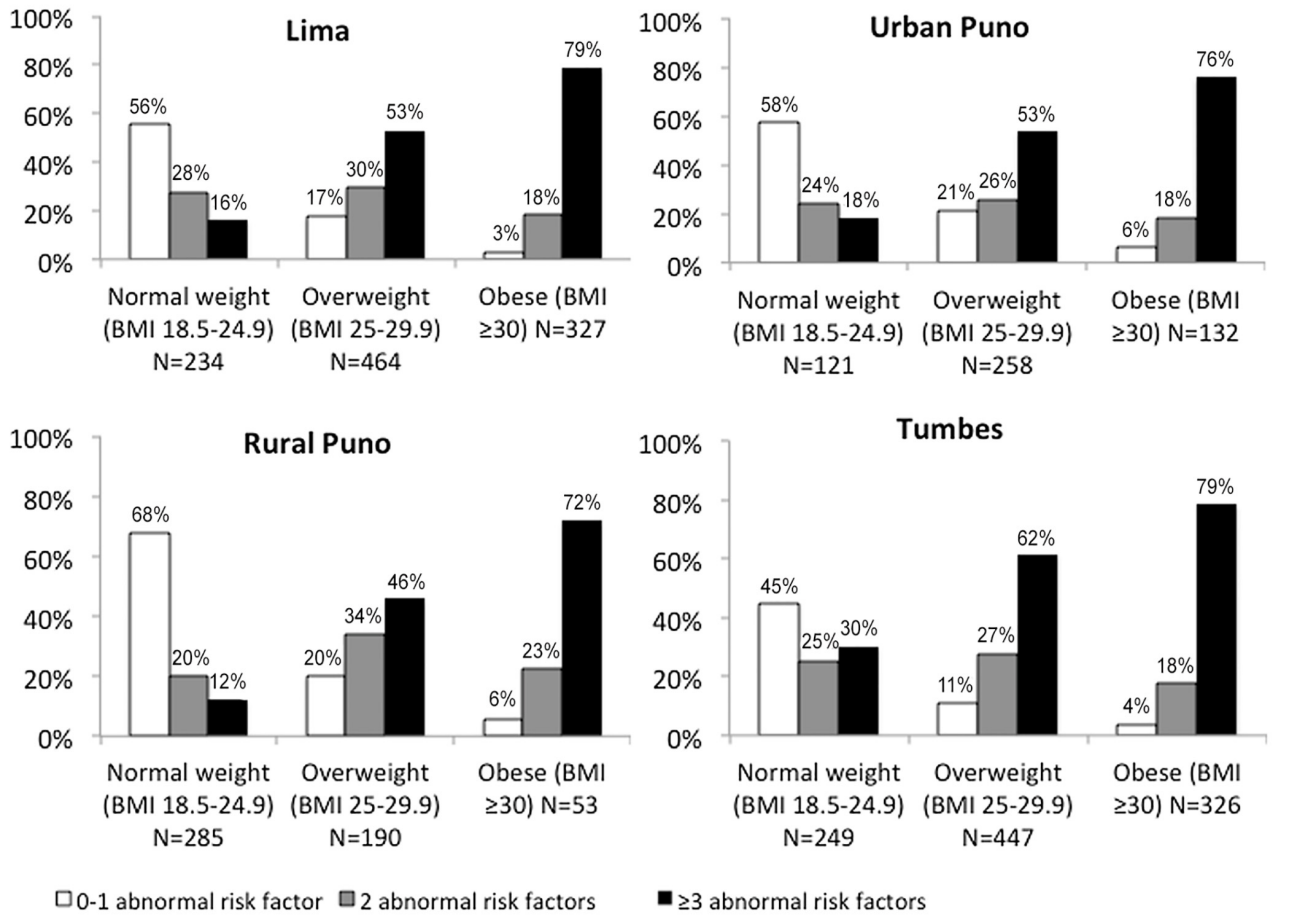
Prevalence of metabolic status by body mass index (normal weight, overweight and obese) by site. Abbreviations: BMI, body mass index (calculated as weight in kilograms divided by height in meters squared).

Fig 1 shows the distribution of number of risk factors by normal weight, overweight and obese separated by site. As BMI increased, the number of risk factors increased. Overall, 19.0% of the normal weight group compared to 54.9% of the overweight and 77.7% of the obese group had ≥3 risk factors (p<0.001), and was consistent at each site.

We next determined if there was a subset of the normal weight group who had multiple metabolic risk factors despite their normal BMI. Overall, there were a total of 2324 (75.3%) participants who were metabolically unhealthy and 16.5% were of normal weight. Within the unhealthy group, compared to the overweight and obese individuals, those who were normal weight were more likely to be older, male, live in rural Puno, have less education level, lower wealth index, and lower physical activity (Table 2). Clinically, they also had lower waist circumference, higher blood pressure, higher HDL-c, lower hypertriglyceridemia, lower impaired fasting glucose, and lower HOMA-IR and hs-CRP levels (Table 2).

Alternatively, we determined if there was a subset of the overweight and obese group who were relatively healthy with either none or 1 metabolic risk factor. Overall, there were 762 (24.7%) participants who were metabolically healthy, and of those, 29.3% were overweight and 4.3% were obese (Fig 1). Within the healthy group with none to 1 metabolic risk factor, compared to the normal weight individuals, those who were overweight and obese were more likely to be younger, female, live in Tumbes, have higher education level, and higher wealth index. Clinically, they also had higher waist circumference, lower blood pressure, and higher HDL-c (Table 2).

### Prevalence of normal weight and metabolic status

The multivariate model using data from normal weight individuals, the metabolic unhealthy profile was more prevalent in those with older age groups (55–64 years and ≥65 years), females, those living in Tumbes, higher education level, and highest tertile of wealth index (Table 3). After adjusting for waist circumference, the oldest age group, female sex, and the highest tertile of wealth index had a higher prevalence of the unhealthy profiles (Table 3).

**Table 3:** Unadjusted and multivariable-adjusted prevalence ratios of the metabolic unhealthy profile associated with socio-demographic and behaviors variables among normal weight individuals (N = 889).

	Unadjusted		Multivariable- adjusted^a^		Multivariable-adjusted^a^ with waist circumference	
	Prevalence ratios (95% CI)					
**Age groups**						
35–44 years	1	Reference	1	Reference	1	Reference
45–54 years	1.13	(0.86–1.48)	1.15	(0.88–1.50)	1.05	(0.81–1.35)
55–64 years	**1.35**	**(1.05–1.76)**	**1.42**	**(1.10–1.85)**	1.26	(0.98–1.62)
≥65 years	**1.57**	**(1.24–1.98)**	**1.68**	**(1.29–2.18)**	**1.43**	**(1.11–1.85)**
**Sex**						
Female (vs. male)	**1.37**	**(1.18–1.60)**	**1.39**	**(1.19–1.63)**	**1.69**	**(1.45–1.97)**
**Site**						
Lima	1	Reference	1	Reference	1	Reference
Urban Puno	0.96	(0.74–1.23)	0.86	(0.66–1.13)	0.90	(0.70–1.16)
Rural Puno	**0.73**	**(0.59–0.91)**	0.87	(0.67–1.11)	0.98	(0.78–1.24)
Tumbes	**1.25**	**(1.04–1.50)**	**1.33**	**(1.09–1.62)**	1.11	(0.93–1.34)
**Education**						
None or primary	1	Reference	1	Reference	1	Reference
Secondary	**0.75**	**(0.62–0.90)**	0.94	(0.76–1.16)	0.92	(0.75–1.13)
Superior	1.05	(0.86–1.28)	**1.31**	**(1.01–1.69)**	1.24	(0.97–1.59)
**Wealth Index**						
Lowest	1	Reference	1	Reference	1	Reference
Middle	1.07	(0.89–1.29)	1.12	(0.92–1.35)	1.01	(0.84–1.21)
Highest	**1.33**	**(1.12–1.60)**	**1.38**	**(1.11–1.71)**	**1.24**	**(1.02–1.51)**
**Daily smoking**						
Current daily smoking (vs. no)	1.07	(0.75–1.53)	0.97	(0.68–1.38)	1.07	(0.77–1.49)
**Alcohol drinking**						
Hazardous drinking (vs. no)	0.84	(0.66–1.06)	1.14	(0.89–1.48)	1.04	(0.81–1.32)
**Leisure time physical activity**						
Moderate/highly active (vs. low)	0.84	(0.56–1.25)	0.81	(0.55–1.18)	0.82	(0.57–1.18)
**Transport-related physical activity**						
Moderate/highly active (vs. low)	**0.72**	**(0.62–0.84)**	0.86	(0.73–1.02)	0.91	(0.78–1.06)
**Waist circumference, per 5cm**	**1.29**	**(1.22–1.36)**	—		**1.30**	**(1.23–1.37)**

Bolded when p-value < 0.05

a. Adjusted for all the variables except for waist circumference in column 2 and all of the variables, including waist circumference in column 3.

### Prevalence of overweight/obese and metabolic status

The metabolically healthy obese profile was more prevalent in those individuals living in urban and rural Puno, those with secondary or superior education, hazardous drinkers, and those with high levels of physical activity; whereas, older age groups, male sex, living in Tumbes and waist circumference had a lower prevalence of the metabolically healthy obese profile. In a multivariate adjustment, living in urban and rural Puno remained significant with a higher prevalence of the healthier profile, while older age groups, male sex, living in Tumbes remained significant with a lower prevalence of the healthier profile. In a multivariate adjustment model that included adjusting for waist circumference, only male sex was associated with a lower prevalence of healthy profile; whereas, living in urban and rural Puno had a higher prevalence of the healthier profile (Table 4).

**Table 4:** Unadjusted and multivariable-adjusted prevalence ratios of metabolic healthy profile associated with socio-demographic and behaviors variables among overweight and obese individuals (N = 2198).

	Unadjusted		Multivariable- adjusted^a^		Multivariable-adjusted^a^ with waist circumference	
	Prevalence ratios (95% CI)					
**Age groups**						
35–44 years	1	Reference	1	Reference	1	Reference
45–54 years	0.85	(0.62–1.16)	0.85	(0.63–1.15)	0.96	(0.73–1.27)
55–64 years	**0.70**	**(0.50–0.98)**	**0.72**	**(0.52–0.99)**	0.87	(0.64–1.19)
≥65 years	**0.60**	**(0.41–0.88)**	**0.60**	**(0.40–0.89)**	0.82	(0.57–1.18)
**Sex**						
Female (vs. male)	**0.52**	**(0.41–0.67)**	**0.53**	**(0.40–0.70)**	**0.40**	**(0.31–0.53)**
**Site**						
Lima	1	Reference	1	Reference	1	Reference
Urban Puno	1.38	(1.00–1.91)	**1.48**	**(1.06–2.08)**	**1.63**	**(1.20–2.23)**
Rural Puno	**1.46**	**(1.01–2.11)**	**1.60**	**(1.10–2.33)**	**1.52**	**(1.04–2.21)**
Tumbes	**0.70**	**(0.50–0.96)**	**0.71**	**(0.51–0.97)**	0.96	(0.70–1.31)
**Education**						
None or primary	1	Reference	1	Reference	1	Reference
Secondary	**1.39**	**(1.04–1.86)**	1.01	(0.74–1.37)	1.08	(0.80–1.44)
Superior	**1.44**	**(1.05–1.97)**	0.84	(0.56–1.25)	0.88	(0.61–1.27)
**Wealth Index**						
Lowest	1	Reference	1	Reference	1	Reference
Middle	1.31	(0.94–1.83)	1.32	(0.95–1.84)	1.50	(1.07–2.11)
Highest	1.28	(0.92–1.78)	1.22	(0.83–1.78)	1.43	(0.98–2.10)
**Daily smoking**						
Current daily smoking (vs. no)	0.80	(0.36–1.80)	0.78	(0.36–1.68)	0.85	(0.41–1.76)
**Alcohol drinking**						
Hazardous drinking (vs. no)	**1.75**	**(1.29–2.36)**	1.11	(0.82–1.50)	1.25	(0.95–1.66)
**Leisure time physical activity**						
Moderate/highly active (vs. low)	1.18	(0.71–1.95)	1.03	(0.64–1.66)	0.87	(0.57–1.33)
**Transport-related physical activity**						
Moderate/highly active (vs. low)	**1.31**	**(1.01–1.73)**	0.93	(0.71–1.23)	0.96	(0.74–1.24)
**Waist circumference, per 5cm**	**0.64**	**(0.58–0.68)**	—		**0.58**	**(0.53–0.64)**

Bolded when p-value < 0.05

a Adjusted for all the variables except for waist circumference in column 2 and all of the variables, including waist circumference in column 3.

## Discussion

### Main findings

In this study, we report a high prevalence of metabolic abnormalities in Peruvian population. Among normal weight Peruvians, over one-third were metabolically unhealthy, as high or higher than estimates reported in developed countries [7, 34]. There were few overweight and obese individuals with a healthy metabolic profile. Therefore, obesity is highly associated with having additional risk factors for CVD in this population.

### Metabolic profiles in the literature

A prior study using the NHANES data found that 30.1% of normal weight Americans were metabolically unhealthy, with a slightly higher percentage (32.8%) in Mexican-Americans [7]. In Canada, one study found that 20% of normal weight people were metabolically abnormal by percentage body fat [34]. Our findings are higher than either of these populations as one-third were metabolically unhealthy (≥2 risk factors) with nearly 20% having ≥3 risk factors. While this is lower than the 54.9% of the overweight and 77.7% of the obese group who had ≥3 risk factors, it is still concerning as Peru was generally considered to be lagging behind many high-income settings in the epidemiological transition from infectious diseases to non-communicable diseases [35]. In addition, there has been a rapid nutritional transition over the past 15 years from undernutrition to over nutrition, which may contribute to the high prevalence of these risk factors [22]. Prevention programs aimed at individuals with a normal BMI, in addition to those who are overweight, are urgently needed, such as screening for elevated fasting cholesterol and glucose.

Prior studies in Peru have noted an increased risk of obesity in females [24, 25], but this is the first study to find that females who are normal weight also have increased risk of being unhealthy in this population. Prior studies in the United States found that older age, males, and those with moderate physical activity were correlated with being normal weight and metabolically unhealthy [7]. Our study also found these factors, as well as living in a high-altitude setting, Puno, had a higher prevalence of the healthier profile. Prior studies have also noted the benefits of high altitude leading to lower weight and CVD rates [36].

The present analysis expands on previous observations in Caucasian populations that those with overweight and obesity are a heterogeneous group with regards to their metabolic risk factors and not all overweight individuals have increased CVD outcomes and mortality. However, our findings are interesting in that less than 5% of those with obesity have metabolic healthy profiles. Most studies in Caucasian populations have found much higher prevalence of metabolically healthy obese profile: from 7% (Finland) to 28% (United Kingdom) and 31.7% (United States) [7–9, 34]. The reason for this difference is unknown. Correlates of the metabolically healthy obese phenotype in the United States are older age, high physical activity, moderate alcohol use and non-Hispanic black ethnicity [7]. We found a higher prevalence with males and living in urban and rural Puno in this small metabolically healthy obese group. A recent meta-analysis suggests that obesity increased all-cause mortality compared to healthy normal weight individuals and that there is not a protective effect of being obese and healthy [8], arguing against the “obesity paradox” [17]. In addition, elevated waist circumference has been found to be predictive of mortality at all levels of BMI [37] as well as predict the development of diabetes [13, 14] and CVD and stroke [16]. The “obesity paradox”, where increased BMI is protective in some chronic conditions and among older adults [17], is also culturally acceptable and a social norm in Peru, especially among women. Our findings are very important, as better public health messaging is needed to counter this belief. The low prevalence of metabolically healthy overweight and obese individuals is important for both health care providers and policy makers to understand that obesity is not a protective trait and in fact obesity is associated with additional risk factors for CVD.

### Associated factors

Regarding the individual components of the metabolic syndrome, all five components had a disturbingly high prevalence—over one-quarter of the population—higher than reported in prior studies in this region [3, 26, 38–40], with the highest prevalence in abdominal obesity (73%) and low HDL-c (66.4%). One study found that exercise programs helped individuals who were metabolically unhealthy and obese become metabolically healthy, yet still obese [41]. Prior studies found the prevalence of overweight and obesity and metabolic syndrome in South America to vary (overweight prevalence 40–69% and obesity prevalence 11–31%) with Brazil being the thinnest and Paraguay having the most obese populations [40]. In that study, the prevalence of obesity in Peru was 16.3%, which is lower than our study where 27.1% are obese. The prevalence of metabolic syndrome in the CARMELA study was between 14–26%, with 18% in Lima [27]. The PREVENCION study in Peru found the prevalence of metabolic syndrome between 17.3%-21.7% in men and 24.0%-25.3% in women depending on which definition was used (American Heart Association or International Diabetes Federation) [26]. Our study, which included HOMA-IR and hs-CRP, found a significantly higher prevalence of metabolic abnormalities with 75.1% having ≥2 abnormal risk factors. When we analyzed the number of metabolic risk factors separately, we found a dose-related response that was consistent across sites and continued when we looked at 4 or more metabolic risk factors (5.5% of normal weight, 25.0% of overweight, and 46.7% of obese individuals had 4 or more metabolic risk factors; p<0.001). One explanation is that there is a strong correlation between BMI and waist circumference in this population and therefore more overweight and obese individuals were likely to have at least one metabolic risk factor: increased waist circumference. However, we found that as BMI increases, the number of additional metabolic risk factors also increases. Elevated HOMA-IR was present predominantly in those who were overweight and obese, consistent with prior studies [6, 18].

Prior studies found more individuals who fell into the metabolically healthy obese category (between 9% to 41%) and found increased mortality in this group, despite varied definitions of metabolic healthy and unhealthy [12]. In our study, the metabolic healthy profile in obese individuals is almost non-existent (less than 5%). Overall, this reflects the epidemiologic transition that is occurring in Peru where non-communicable diseases are becoming more prevalent and the risk factor profile is similar to many developed countries.

### Limitations

This study has some limitations. The definition of metabolic status used in our study was maintained similar to other published studied [8, 10, 11, 21, 32, 42] to facilitate meaningful comparisons. Therefore, our aim was not to assess individual clinical outcomes by BMI status, but the seven risk factors as a group. Therefore, as prior studies have done, we excluded those who were underweight (n = 19), as meaningful comparisons with the other groups, especially with the large number of individuals in the overweight and obese groups, would have been limited by power issues. In addition, waist circumference was one of the metabolic risk factors used previously [8] as abdominal obesity is an important component of the metabolic syndrome [32] but is closely related to BMI in this population. Lastly, there are no well-established definitions of adiposity for Latin America. Therefore, we used Caucasian cut-offs for BMI, which have not been validated in this population to determine which obesity-related cut-offs are most predictive of CVD-related morbidity and mortality in this population, including percentage body fat and waist circumference. BMI cut-offs still allow for internal comparisons as random error is expected to be equally distributed across groups. Prospective studies are needed to determine the risk associated with cardiovascular events in each metabolic group in this population.

## Conclusion

Our study found that most Peruvians with overweight and obesity have additional risk factors for CVD, as well as a majority of those with a healthy weight. This number is only going to continue to increase unless national prevention programs are put in place to modify the behavioral and clinical risk factors, such as those proposed by the American Heart Association or simply exercise alone [39, 41]. Prevention programs aimed at individuals with a normal BMI, in addition to those who are overweight and obese, are urgently needed, such as screening for elevated fasting cholesterol and glucose. Understanding the differential metabolic responses to body size, its long-term consequences on hard outcomes and their potential to improve cardiovascular disease screening tools remain as areas of major challenges for cardiovascular disease prevention in low- and middle-income country settings.
